# It Came From Beneath: Detecting and Mitigating Vapor Intrusion

**DOI:** 10.1289/ehp.124-A141

**Published:** 2016-08-01

**Authors:** Rachel Cernansky

**Affiliations:** Rachel Cernansky is a freelance journalist in Denver, Colorado, covering science, health, and the environment. She has written for publications including *Yale Environment 360*, *Nature*, *Civil Eats*, and *The New York Times*.

Indoor exposure to naturally occurring radon gas has rocketed into public awareness since the 1980s, but now a similar, albeit lesser-known form of indoor pollution is gaining attention of its own. That form is vapor intrusion, the migration of volatile chemicals from groundwater and soil into buildings above them.

**Figure:**
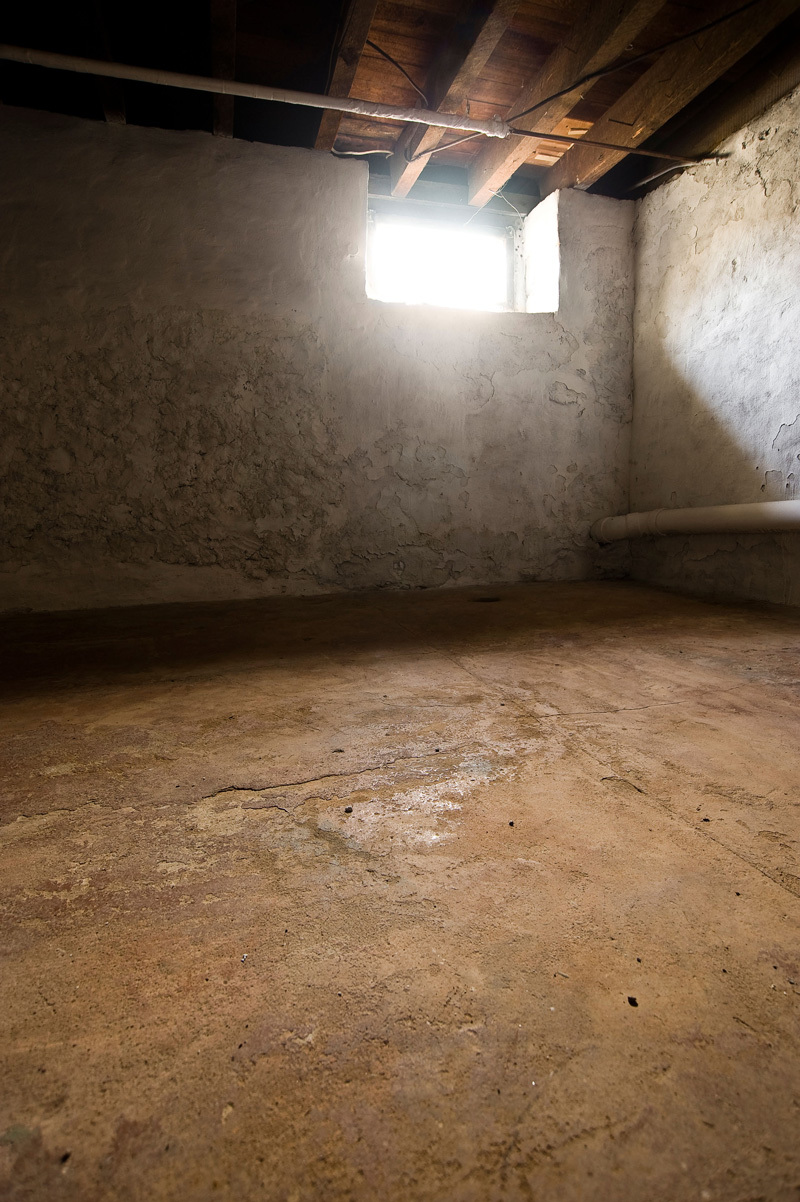
Although indoor air quality has been a topic of intense public health inquiry for many years, it’s only been relatively recent that vapor intrusion has been recognized as a source of indoor air contaminants. Today researchers are searching for better ways to measure and control volatile chemicals that can enter buildings from the soil and groundwater beneath them. © JG Photography/Alamy Stock Photo

There are four main sources of vapor intrusion: industrial sites, military sites, dry cleaners, and gas stations—all locations that produced or heavily used solvents, degreasers, and other volatile chemicals, or that still do. Few experts feel comfortable estimating the scale of vapor intrusion in the United States. But Kelly Pennell, an engineering associate professor at the University of Kentucky Superfund Research Center, says, “Almost every urban environment we have has some kind of historical contamination associated with it.” If volatile chemicals are present as contaminants, then vapor intrusion may be a concern.

“No one really knew [vapor intrusion] had the potential to have as many impacts as it does until the late 1990s and even into the 2000s,” says Dave Folkes, a Colorado-based engineer and senior principal at Geosyntec Consultants. “There was a lot of fear, unknowns, uncertainty because it was very new science, and we really didn’t know much about it. We were all running around trying to learn how to do things quickly.”

Today, Folkes says, the field is more mature; there are tools for assessing and mitigating vapor intrusion, and regulations governing how to deal with it. With that maturation has come a more nuanced understanding of how vapor intrusion occurs and the health risks it can pose. It has also raised new questions.

“Vapor intrusion is a complex problem that requires the integration of multiple disciplines to address the issue,” says William Suk, director of the Superfund Research Program of the National Institute of Environmental Health Sciences (NIEHS), which funds a number of vapor intrusion projects. “By looking at the problem from a health perspective and an engineering perspective, scientists are working to understand how to detect chemicals and predict exposure, identify health concerns, and mitigate the problem.”

## Chemicals of Concern

The chemicals of greatest concern with respect to vapor intrusion are chlorinated solvents such as trichloroethylene (TCE), commonly used as a metal degreaser, and tetrachloroethylene (also known as perchloroethylene, or “perc”), another degreasing agent widely used in dry cleaning. Petroleum-derived compounds—for instance, what you would see with gasoline and motor oil contamination—are considered somewhat less of a threat than TCE and perc, says Eric Suuberg, co-director of the Superfund Research Program at Brown University. That’s because these compounds are much more prone to biodegradation in the soil, compared with more stable chlorinated solvents.

Long-term inhalation exposures to perc have been linked to negative impacts on cognitive and motor functions, numerous types of cancer, damage to the liver and kidney, and adverse immune and hematologic effects. There is also evidence that even low-level exposures over time can cause neurological and neurobehavioral effects.^1^

In 2011 researchers reported associations between elevated rates of risky behaviors in youth (such as smoking and drug use) and exposure to high levels of perc in drinking water during gestation and early childhood.^2^ Another recent retrospective study found an association between increased risk of epilepsy and certain cancers (especially cervical cancer) in adults following early-life exposures to perc-contaminated drinking water.^3^ There is also evidence suggesting that perc in drinking water may cause birth defects, although the findings are far from conclusive.^1^

Exposures to TCE, meanwhile, are known or suspected to cause several types of cancer in humans. Epidemiological and animal studies have linked drinking-water exposures to decreased body weight and markers of liver and kidney damage, and inhalation exposures can cause neurological, immunological, reproductive, and developmental effects.^4^ There is also evidence that TCE may cause structural defects in the developing fetal heart.^5^

In 2011 the EPA updated its health hazard assessment for TCE and lowered the reference values used to characterize acceptable lifetime oral and inhalation exposures.^4^ This was largely driven by concerns about the effects of TCE or its metabolites on fetal heart development. But Wendy Heiger-Bernays, an associate professor of environmental health at the Boston University School of Public Health Superfund Research Program, says there is significant controversy about the very low values, which are based on organ, cellular, and molecular studies in animals.

There are concerns among some researchers and regulators that the evidence for cardiac effects is not yet strong enough or consistent enough to warrant restructuring risk management policies.^5^ ^,^ ^6^ “The controversy stems from the recognition that these are low concentrations, there are many vapor intrusion sites and homes and businesses that are affected, and the financial costs and challenges for risk communication are large,” Heiger-Bernays explains.

## Legacy of Contamination

Certain well-known contaminated sites in the United States have yielded considerable insight into how vapor intrusion works. One of these is a manufacturing facility in Endicott, New York, which IBM operated until the 1980s. The groundwater below about 320 acres of the downtown and residential areas became—and to some extent remains—contaminated with TCE, perc, and other chemicals. An epidemiological study of the Endicott site found an association between cardiac defects and maternal residence in areas where both TCE and perc vapor intrusion was a problem, as well as associations between low birth weight and fetal growth restriction among mothers who lived in an area affected primarily by TCE.^7^

Another notable site is located in Denver, Colorado, where for decades the Redfield manufacturing facility made rifle scopes and similar products. It became a focus for state agencies in the late 1990s, after groundwater beneath the site was found to be contaminated with cleaning solvents, including TCE.^8^ The Redfield site was one of the first major vapor intrusion sites identified in the United States, and it quickly illustrated how relatively small-scale pollution can have a profound environmental impact.

“The Redfield site was a very small manufacturing facility that simply removed the grease from rifle scope shells,” says Folkes, who served as project manager for the investigation and mitigation program at the site. “The [leaking degreaser unit] was probably something like four feet by eight feet in size, and it resulted in a groundwater plume that goes about two miles.”

Meanwhile, the detection of elevated TCE levels at Google’s offices in Mountain View, California, several years ago illustrates how the potential for vapor intrusion can easily shift into a reality. According to Lenny Siegel, executive director of the nonprofit California-based Center for Public Environmental Oversight, the buildings were built on a site known to be contaminated, but sampling confirmed that the facility’s HVAC system prevented vapor intrusion. Indoor levels of TCE began rising, however, when problems with the HVAC system and an opening in the slab altered the air pressure in the building. Once these issues were corrected, TCE concentrations returned to acceptable levels.^9^

Apart from well-documented cases such as these, it has become increasingly clear there are innumerable small plumes around the country. “Anywhere you poke a hole in New York City, or any big city, you’re going to find old dry cleaning contamination,” says Siegel.

Larry Schnapf, a New York–based environmental attorney, says he’s aware of groundwater plumes throughout the city for which there is no official documentation, because a buyer or seller has discovered it during a property transaction—deals that were then called off without ever making the discovery public. “I’ve seen samples from deals that didn’t happen because the buyer saw the results and said, ‘I don’t want to buy this pig in a poke,’” he says. “So the buyer doesn’t report it. The seller’s not going to report because they don’t have a deal anymore, and [the contamination] just sits there.”

It can be difficult to characterize vapor intrusion, partly because there are other sources of volatile chemicals in indoor air. Storing gasoline indoors or in an attached garage, use of certain commercial degreasing agents, and even bringing home freshly dry-cleaned clothing can elevate indoor levels of chemicals associated with vapor intrusion, Suuberg says.

**Figure:**
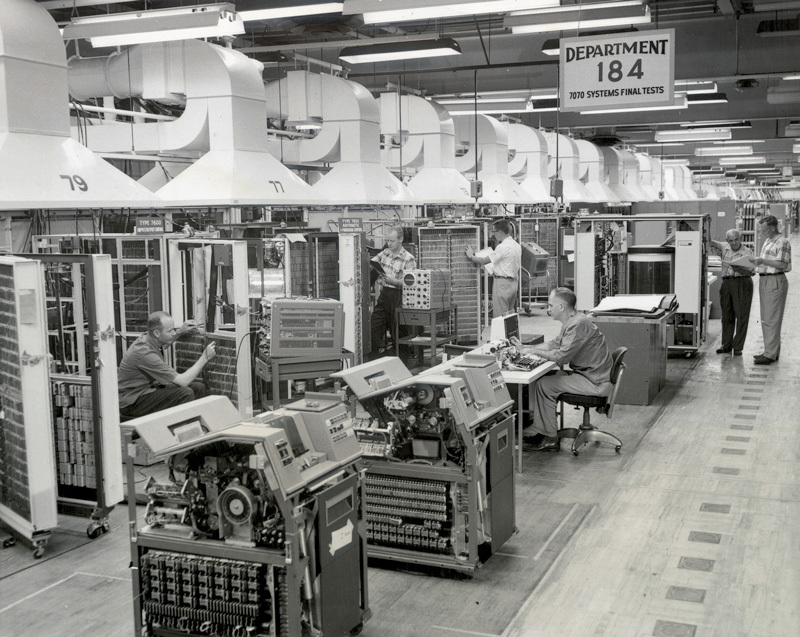
Vapor intrusion is often associated with contaminated groundwater. The widespread use of volatile chemicals has resulted in innumerable groundwater plumes across the United States, some documented, some not. IBM’s manufacturing plant in Endicott, New York, seen here circa 1958, is one of the better-studied sites. © George Rinhart/Getty Images

That means simply detecting a chemical of concern in an indoor air sample is not enough to diagnose a vapor intrusion problem. “All of these kinds of resident-controlled sources need to, in some fashion, be accounted for or eliminated from consideration when a building is being assessed for a vapor intrusion hazard,” Suuberg says.

Many researchers are exploring how to quantify vapor intrusion into buildings, given that levels of indoor air contaminants vary significantly over time, with some of the largest fluctuations coming with changes in weather or in season.^10^ “That is really where the heart of the field is right now—trying to sort out just how densely in time you have to take data in order to support sound regulatory decisions,” Suuberg says. “And no one has an answer.” That lack of certainty is one reason why there is not yet a generally agreed-upon method for characterizing indoor air contaminants.

## Mitigating the Problem

The good news is that once vapor intrusion is discovered—and acknowledged—mitigating the problem is often relatively straightforward. Immediate steps to reduce exposure include setting up portable air purifiers, says Paul Locke, the assistant commissioner for waste site cleanup at the Massachusetts Department of Environmental Protection. He says his agency keeps several such units on hand for use as a stopgap measure in residential areas that are found to be contaminated. “We can take those initial steps very, very quickly—in a matter of days,” Locke says.

**Figure:**
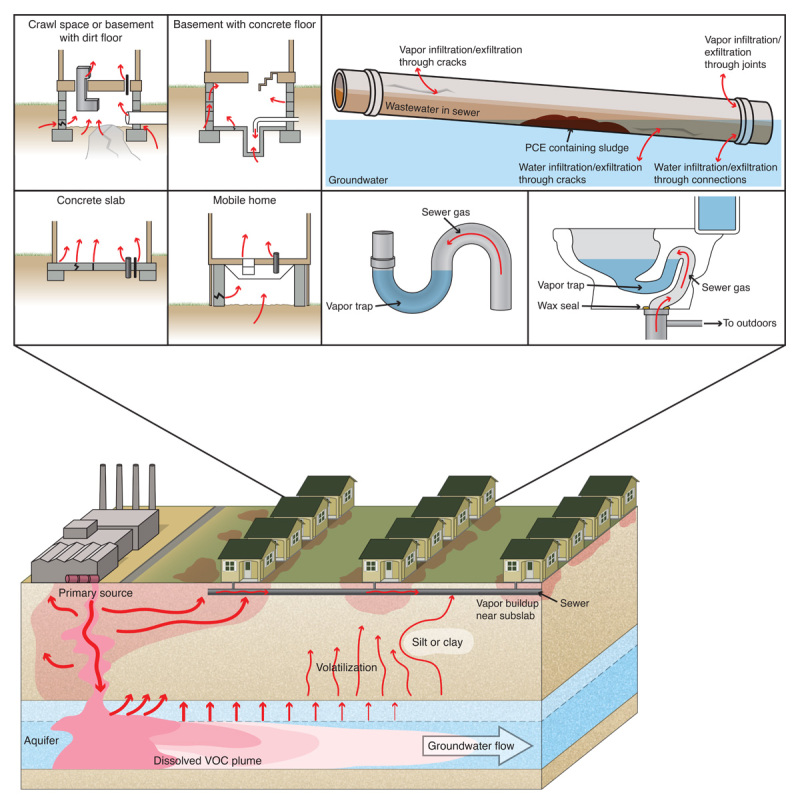
When chemicals are spilled or dumped on the ground, vapors can form and travel far through the soil. Under the right conditions, chemicals that make their way into groundwater can travel miles from the original source, following the path of least resistance. Vapors may enter homes by any of a number of pathways, including cracks, seams, or openings in a building’s foundation or floor; expansion joints in a concrete slab; or fractured rock exposed in a crawlspace. Newer research indicates sewer pipes also may contribute to vapor intrusion. Vapors can enter sewer pipes through cracks or improperly fitted joints, and sewage itself may contain volatile chemicals that were dumped either legally or illegally into wastewater. Leaky or malfunctioning plumbing could allow vapors in a sewer line to enter a home. Illustration: Jane Whitney for EHP. Adapted from EPA (2008),^11^ Pennell et al. (2013),^16^ and Dawit Bekele and Ravi Naidu (unpublished)

For a long-term solution, in many cases the answer is to install a vapor intrusion mitigation system. Traditional mitigation systems consist of a layer of gravel below the floor slab, which produces void spaces for the gas to travel, and riser pipes that collect and vent the vapors. These systems work by depressurizing the soil beneath a building to intercept and then vent the vapors outdoors, either passively or with the use of fans.^11^

**Figure:**
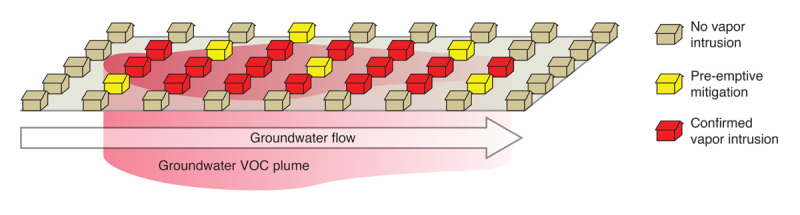
If indoor air samples in a few homes indicate vapor intrusion, the likelihood of intrusion can be calculated for homes of the same era with similar construction, even if no sampling data are available. In these cases, preemptive vapor mitigation may be recommended. Illustration: Jane Whitney for EHP. Adapted from EPA (2015)^18^

According to Folkes, most new developments have focused on standard system approaches that simply use better materials or operate more sustainably (for instance, automated systems). He says the most advanced and promising alternative to traditional systems is the concept of the “aerated floor,” pioneered by a company called Pontarolo Engineering. This company developed recycled plastic forms known as Cupolex® to replace the gravel layer in the bottom of the concrete slab.^12^ Folkes says the larger void spaces produced by Cupolex® move air more easily than gravel, providing more efficient venting and allowing the use of much smaller fans.

Some experts and regulators support the approach of installing a mitigation system at any new construction site near areas with known groundwater contamination. That reduces potential risks and costs from day one, rather than having to assess and mitigate—or worry about overlooking—problems later. (This preemptive strategy echoes recommendations from a radon workgroup led by the American Lung Association. In 2015 that group recommended that state and local building codes be updated to require radon risk-reduction measures such as radon mitigation systems.^13^)

For Entanglement Technologies CEO Tony Miller, the ultimate vision is for sampling technology to become a standard part of construction and to pair it with a vapor intrusion mitigation system. His company’s Autonomous Rugged Optical Multigas Analyzer, or AROMA, was designed to be a portable, easy-to-use sensor that can take many measurements quickly and at different points within a building, potentially reducing assessment costs.^14^ Currently in the prototype stage, with field trials planned, the system can, in theory, identify the source of vapors more easily and much more quickly than some of the current techniques.

There is also evidence that amending contaminated soil with organic matter such as biochar (a form of charcoal) may mitigate some of the risks.^15^ “Where you have uncontaminated mineralized clay soil or organic matter present overlaying a contaminated groundwater plume, the soil media provides a very strong binding surface for volatiles,” says Ravi Naidu, director of the Global Centre for Environmental Remediation at Australia’s University of Newcastle. He explains that organic matter can bind volatile chemicals and mitigate the threat. By the same token, he says, the amount of clay, organic matter, and secondary minerals present in soil is a key factor in predicting the risk of intrusion.^15^

## Unconventional Routes

But it turns out that vapors don’t always enter buildings through the soil. In a case study published in 2013, Pennell and coauthors demonstrated that perc entered a home through a sewer line, probably through a faulty wax seal on the toilet.^16^ Once the toilet connection was sealed, the levels of perc decreased to acceptable levels. “When we repair plumbing systems, we usually focus on water, but we don’t think about vapors leaking unless it becomes an odor nuisance,” she says.

Pennell’s research group is now trying to develop a model to better understand how vapors are transported through sewers and into indoor air. She explains that chemicals can enter sewer systems in the wastewater itself, a result of both legal and illegal discharges. Contaminated groundwater and vapors also can enter through cracked or otherwise deteriorated sewer lines. (She points out that aging infrastructure is part of a larger challenge faced by municipalities, with groundwater entry into sewer lines a well-recognized problem.^17^)

The possibility of vapors traveling along unconventional routes to get inside buildings has presented a stumbling block for researchers, who are trying to figure out how to incorporate this extra variable into existing methods for predicting how vapor will travel.

**Figure:**
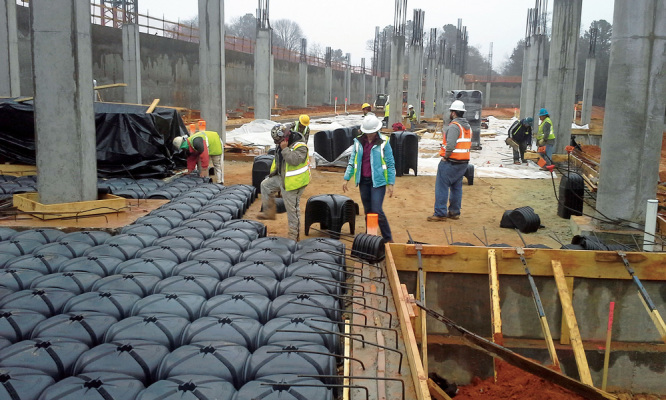
These workers are installing an aerated floor. Similar to a traditional vapor mitigation system, an aerated floor enables vapor rising beneath a building to be vented safely outdoors. Image courtesy of Geosyntec Consultants

“We have these rules of thumb that we’ve been developing over the last ten to fifteen years that help us investigate. For example, we normally don’t see impacts more than about a hundred feet beyond the edge of a groundwater plume,” says Folkes. “But there’s been concern that what we call preferential pathways—which might be a sewer, or maybe a real high-permeability gravel layer or … fractures in rock—could cause vapors to go further, faster, and in higher concentrations than our rules of thumb would say.”

While Folkes does not think this typically occurs, in certain situations it can be a concern. For example, he says that at a research house owned by Arizona State University, a foundation drain pipe was found to be connected to a storm sewer containing contaminated groundwater. This effectively served as a direct pipeline of vapors into the house, Folkes explains.

Because contaminated groundwater is fairly common in developed cities, vapor intrusion is far more common than most people realize, Pennell says. But if there is a silver lining, it is that the growing awareness of vapor intrusion has highlighted the importance of indoor air quality and its effect on human health.

“By installing mitigation systems, limiting the use of consumer products that contain [volatile chemicals], remediating contaminated groundwater, and understanding how building ventilation systems impact indoor air quality,” Pennell says, “we can protect against many of the environmental health risks and promote healthier communities.”

## References

[1] EPA Integrated Risk Information System (IRIS).. https://cfpub.epa.gov/ncea/iris/iris_documents/documents/subst/0106_summary.pdf.

[2] Aschengrau A Affinity for risky behaviors following prenatal and childhood exposure to tetrachloroethylene (PCE)-contaminated drinking water. Environ Health 10 102 2011, doi:10.1186/1476-069X-10-102 22136431 PMC3268745

[3] Aschengrau A Long-term health effects of early life exposure to tetrachloroethylene (PCE)-contaminated drinking water: a retrospective cohort study. Environ Health 14 36 2015, doi:10.1186/s12940-015-0021-z 25889838 PMC4397674

[4] EPA Integrated Risk Information System (IRIS).. https://cfpub.epa.gov/ncea/iris/iris_documents/documents/subst/0199_summary.pdf.

[5] Chiu WA Human health effects of trichloroethylene: key findings and scientific issues. Environ Health Perspect 121 3 303 311 2013, doi:10.1289/ehp.1205879 23249866 PMC3621199

[6] IDEM 2016 Screening Levels Table Now Available.. http://www.in.gov/idem/landquality/files/risc_screening_table_2016_announce.pdf.

[7] Forand SP Adverse birth outcomes and maternal exposure to trichloroethylene and tetrachloroethylene through soil vapor intrusion in New York State. Environ Health Perspect 120 4 616 621 2012, doi:10.1289/ehp.1103884 22142966 PMC3339451

[8] Brown Group Retail Redfield Site: Environmental Fact Sheet (September 2006).. http://www.redfieldsite.org/pdfs/redfield_site_environmental_fact_sheet_-_september_2006.pdf.

[9] Welt SB, Bice NT Indoor Air Sampling Report. Former Fairchild Buildings – Google Quad, 369, 379, 389 and 399 North Whisman Road and 468 Ellis Street.. https://www.documentcloud.org/documents/612000-369-399-n-whisman-ia-report-22february13-final.html.

[10] Schumacher B, et al. Fluctuation of Indoor Radon and VOC Concentrations Due to Seasonal Variations. EPA/600/R-12/673. Washington, DC:U.S. Environmental Protection Agency (2012). Available: https://cfpub.epa.gov/si/si_public_record_report.cfm?dirEntryId=247212 [accessed 22 July 2016]

[11] EPA Engineering Issue: Indoor Air Vapor Intrusion Mitigation Approaches.. http://nepis.epa.gov/Adobe/PDF/P100AE72.pdf.

[12] Cupolex [website]. Vaughan, Ontario, Canada:Pontarolo Engineering (2007). Available: http://www.pontarolo.ca/html/cupolex.shtml [accessed 22 July 2016]

[13] EPA The National Radon Action Plan: A Strategy for Saving Lives.. https://www.epa.gov/sites/production/files/2015-11/documents/nrap_guide_2015_final.pdf.

[14] Environmental Sensing [website]. Burlingame, CA:Entanglement Technologies, Inc. (2016). Available: http://www.entanglementtech.com/environmental.html [accessed 22 July 2016]

[15] Bekele DN Influence of soil properties on vapor-phase sorption of trichloroethylene. J Hazard Mater 306 34 40 2015, doi:10.1016/j.jhazmat.2015.12.002 26686522

[16] Pennell KG Sewer gas: an indoor air source of PCE to consider during vapor intrusion investigations. Ground Monit Remed 33 3 119 126 2013, doi:10.1111/gwmr.12021 PMC3740581 23950637

[17] ASCE Report Card for America’s Infrastructure.. http://infrastructurereportcard.org/a/documents/2013-Report-Card.pdf.

[18] EPA OSWER Technical Guide for Assessing and Mitigating the Vapor Intrusion Pathway from Subsurface Vapor Sources to Indoor Air.. https://www.epa.gov/vaporintrusion/technical-guide-assessing-and-mitigating-vapor-intrusion-pathway-subsurface-vapor.

